# Expanding *Nature Notes*—A space for ecological observations, new species, and species records

**DOI:** 10.1002/ece3.9215

**Published:** 2022-09-12

**Authors:** Gareth B. Jenkins, Diogo B. Provete, AJ Harris

**Affiliations:** ^1^ John Wiley & Sons Ltd Oxford UK; ^2^ Instituto de Biociências Universidade Federal de Mato Grosso do Sul Campo Grande Brazil; ^3^ Gothenburg Global Biodiversity Centre Göteborg Sweden; ^4^ Key Laboratory of Plant Resources Conservation and Sustainable Utilization, South China Botanical Garden Chinese Academy of Sciences Guangzhou China

Back in July 2020, *Ecology & Evolution* launched a new type of article category entitled “Nature Notes” (Moore et al., 2020). This was our attempt to reinvigorate the publication of descriptive natural history studies, which we felt were becoming harder to publish in a landscape that increasingly focuses on hypothesis‐driven science above all else. We wanted to see unusual and undescribed accounts of ecology and behavior, and the success of the article type to date has been encouraging. We have published 112 Nature Notes in the two years since the article type has been active, and there have been other launches in our fields for similar manuscript types (Powers et al., 2021). Submissions continue to grow, and these papers tend to attract significant attention from the news media (e.g., Bastos et al., 2021). However, recent discussions among the Editorial Board have highlighted a bias within these papers; that they are overwhelmingly focused on behaviors and new observations of a few select, previously known to science taxa, such as charismatic mammals and/or birds. Of the 112 Nature Notes we have published, 99 were zoological, 11 were botanical, and only two described observations of prokaryotes. In addition, they tend to be ecological rather than evolutionary. We expect that the emphasis on behavior and observation in the first editorial introducing this article type is the reason for this, though we did not mean to be exclusive of descriptions of new species, which embody taxonomic and evolutionary themes. Thus, we are writing in order to remedy this in the hopes to attract papers including new species and species observed in habitats or geographic areas where they were previously unknown to occur.

We are issuing both an open call for Nature Notes manuscripts that go beyond zoological observations and are especially seeking emphasis on botanical, fungal, eukaryotic microbes, and prokaryotic organisms. New species descriptions and new records are particularly welcome. Such papers are increasingly important given that species are a fundamental unit in ecological and evolutionary research, that the impacts of climate change are leading to local and global extirpations and the loss of species via hybridization following human‐mediated invasions. We feel that acceptance of such manuscripts represents the role we can play in the calls for cataloging biodiversity (e.g., Ehrlich & Wilson, 1991). To this end, we are also keen to ensure that studies in cryptic diversity and on taxonomic reorganizations or reports at levels above species have a broad outlet for publication beyond taxa‐specific journals.

Although we remain happy to receive manuscripts that do not adhere to any specific rules or formatting rules, we strongly recommend that the diagnosis of a new taxon comprises a separate subheading and a list of synonyms with authorities and sources as/if required by the applicable nomenclatural code (please see our Author Guidelines for further details). Moreover, the diagnosis should be done by demonstration; this is to say that true differential diagnosis should be provided that specifies the features that serve to differentiate the new taxon from all others at the rank under consideration rank (or, alternatively, at the within the geographical area of the new taxon).

We further require that all sequence data be deposited in GenBank (Clark et al., 2016; https://www.ncbi.nlm.nih.gov/genbank/) at NCBI (https://www.ncbi.nlm.nih.gov/) even if not required by the relevant code, and pictures and/or micrographs of the organism (ideally of the holotype or type if one is required) should be included in the main text of the manuscript. In addition to making sequence data public, alignments and trees, which are derived through heuristics and usually cannot be reproduced exactly a second time by having the raw data alone, should also be made publicly available through an open repository such as Dryad (https://datadryad.org/), which has fees that can be paid by Ecology and Evolution if the manuscript is accepted.

As always, we continue to apply our author‐friendly criteria to evaluation of these Nature Notes manuscripts, and we look forward to working with authors to get them published.
